# MEOX1: a novel druggable target that orchestrates the activation of fibroblasts in cardiac fibrosis

**DOI:** 10.1038/s41392-021-00842-7

**Published:** 2021-12-24

**Authors:** David Schumacher, Fabian Peisker, Rafael Kramann

**Affiliations:** 1 Institute of Experimental Medicine and Systems Biology, University Hospital Aachen, RWTH Aachen University, Aachen, Germany; 2 Department of Anesthesiology, University Hospital Aachen, RWTH Aachen University, Aachen, Germany; 3 Department of Internal Medicine, Erasmus Medical Center, Erasmus University, Rotterdam, The Netherlands; 4 Department of Nephrology and Clinical Immunology, University Hospital Aachen, RWTH Aachen University, Aachen, Germany

**Keywords:** Cardiology, Target identification

Single-cell genomic technology has been used to identify MEOX1 as a potential cell-specific, druggable target in cardiac fibrosis. To this effect, a landmark study was recently published in *Nature* by Alexanian and colleagues.^1^

Fibrosis is a common response to chronic repetitive injury to vital organs and considered an important therapeutic target to slow down, inhibit, or reverse progression of organ failure. While there is broad interest in developing novel antifibrotic therapeutics, currently, only nintedanib and pirfenidone have been approved to treat fibrosis for one disease entity; idiopathic pulmonary fibrosis.^2^ Both drugs interfere with pro-fibrotic growth factor signaling. Various other anti-fibrotic approaches are currently under investigation. These include small molecules or antibodies interfering with different cytokines involved in fibrosis, senolytic drugs, drugs targeting metabolic changes and macrophage-fibroblast crosstalk and chimeric antigen receptor T-cell (CAR-T) therapy targeting active fibroblasts.^2^ However, none of these approaches have brought anti-fibrotic therapeutics to the clinic and novel therapeutics are still urgently needed.

Recent approaches stemming from targeted inhibition of epigenetic signaling proteins, belonging to the bromodomain and extra terminal domain (BET) family, have shown promising results in preclinical studies of cardiac disease.^3^ BET inhibition suppressed inflammation and fibrosis in models of heart failure. Last year BETonMACE, the first clinical trial investigating BET inhibitors in patients with recent acute coronary syndrome and type 2 diabetes, failed to show a benefit of BET inhibition for cardiovascular death, nonfatal myocardial infarction, or stroke as primary outcome.^3^ However, further analysis of the data was published this year and revealed that BET inhibition was associated with fewer hospitalizations for heart failure in patients with type 2 diabetes and recent acute coronary syndrome.^3^ BET inhibitors broadly affect several cell types throughout the body. Besides reducing fibrosis, they are likely to disrupt many other critical cellular functions. This might explain the failure of the primary outcome of the BETonMACE study. The anti-fibrotic effects of BET inhibitors are promising for the development of new therapeutics in cardiovascular disease. However, side effects associated with BET inhibitors remain a challenge. Therefore, more research is needed to dissect the molecular mechanisms involved in BET inhibition and to develop targeted, cell-specific therapies.

Recently, Alexanian et al. utilized single-cell sequencing technology to uncover stress-activated signaling cascades involved in fibroblast activation in a murine model of heart failure.^1^ Since the preclinical studies of cardiac disease models showed strong reduction in fibrosis after BET inhibition, they used the BET inhibitor JQ1 for a temporally controlled perturbation of transcription signaling. The molecular mechanisms critical for the progression and reversal of cardiac fibrosis were thereby identified. JQ1 treatment in mice undergoing transverse aortic constriction (TAC) resulted in reversion of activated fibroblasts into a less active state, comparable to fibroblasts of sham-operated mice with subsequently improved cardiac function. Withdrawing JQ1 therapy shifted the fibroblasts back to a pro-fibrotic activation status, demonstrating the transcriptional reversibility of the stress-induced signature with BET inhibitors.

**Fig. 1:**
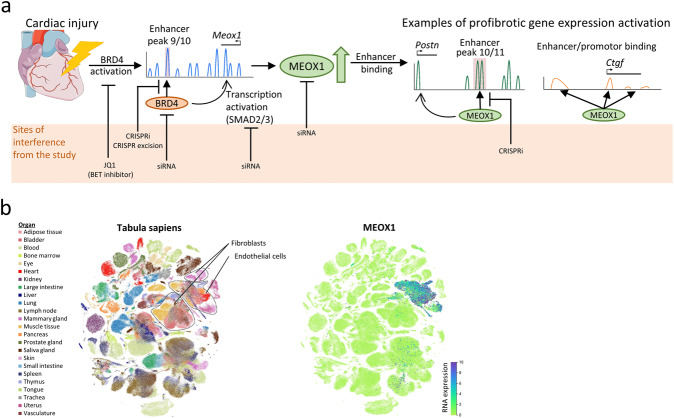
**a** Schematic step-by-step overview of the mechanisms leading from cardiac injury to activation of pro-fibrotic gene expression in fibroblasts, as depicted by Alexanian et al. Sites of experimental interference used to clarify critical regulatory elements are highlighted. (heart graphic source: smart.servier.com) **b** Single-cell RNA expression of MEOX1, including 24 different organs of origin. The figure was generated using the single-cell dataset from the tabula sapiens consortium^6^

The integration of single-cell RNA sequencing data with data from a single-cell assay for transposase-accessible chromatin sequencing (scATAC-seq) from the same hearts, enabled Alexanian et al. to follow the transcriptomic switch and underlying chromatin accessibility changes after TAC. This process could reversibly be attenuated by JQ1 treatment at single-cell resolution (Fig. 1a). In fibroblasts, the scATAC-seq data revealed the transcription factor (TF) Mesenchyme homebox 1 (MEOX1) as one of the TFs with the strongest increase in activity after TAC surgery. In previous studies, MEOX1 has been shown to be involved in organ development, influencing cell cycle states, and inducing senescence in endothelial cells.^4^ In endothelial cells, MEOX1 and MEOX2 have redundant functions.^4^ However, the involvement of MEOX1 in cardiac fibrosis and fibroblast activation has not previously been investigated. The chromatin accessibility data from Alexanian et al. further revealed several accessible cis-regulatory regions near the *Meox1* gene. By combining this data with chromatin immunoprecipitation followed by sequencing and precision nuclear run-on sequencing of fibroblasts stimulated with or without TGFβ, they identified a region located 62–65 kilobases (kb) downstream from the *Meox1* promoter that was marked by an increase in nascent transcription after TGFβ stimulation. They called this region peak 9/10 according to their scATAC-seq data and showed that the specific inhibition of this region by CRISPR–Cas9-based excision or transcriptional interference by CRISPR abolished the TGFβ induced overexpression of MEOX1. SMAD2 and SMAD3, transcriptional mediators of the TGFβ signaling, had several putative binding places in this peak 9/10, as well as in the *Meox1* promoter. Knockdown of SMAD2 and SMAD3 led to a significant reduction of the transcription in peak 9/10 and reduced the transcription of *Meox1*.

Finally, the authors demonstrated that MEOX1 mediates pro-fibrotic functions. In fibroblasts, the knockdown of *Meox1* reduced the differentiation into myofibroblasts after TGFβ stimulation. GO term analysis further suggested a MEOX1-dependent increase in fibroblast proliferation, migration, and cell motility, hallmarks of fibroblast activation. Among the MEOX1-dependent upregulated genes were classic markers of myofibroblasts and fibrosis, such as *Ctgf* and *Postn*. These genes showed increased MEOX1 TF activity at their promoter and proximal regulatory elements (Fig. 1a). Thus, MEOX1 seems to be an important mediator of fibroblast to myofibroblast differentiation. *MEOX1* expression was found to be increased in human heart, lung, liver, and kidney fibroblasts after TGFβ stimulation and upregulated in sequencing data from human cardiomyopathy and idiopathic pulmonary fibrosis. A targeted therapy against MEOX1, therefore, carries potential.

Currently, no official studies are ongoing to develop drugs targeting the transcription factor MEOX 1. Due to significant structural disorder and lack of defined binding pockets for small molecules, transcription factors have been considered undruggable in the last decades, leaving MEOX1-targeting a challenge. Recently, novel strategies for targeting transcription factors have emerged. The modulation of auto-inhibition, proteolysis targeting chimaeras, the use of cysteine reactive inhibitors, and targeting intrinsically disordered regions of transcription factors have all shown promise.^5^ These innovations in drug development highlight the value of cell-type-specific target discovery by novel single-cell genomic approaches and provide a basis for developing a targeted MEOX1 therapy.

Data generated on the single-cell level has a high potential for identifying cell-specific therapeutic targets, essential in reducing side effects. Alexanian’s et al. single-cell data suggest a myofibroblast-specific expression of MEOX1 in cardiac tissue, while data from the Tabula Sapiens consortium also indicates expression of MEOX1 in endothelial cells from various healthy human tissues (Fig. 1b).^6^ However, the activity of MEOX1 as a transcription factor might not directly be regulated by its messenger RNA expression and thus the ATAC-seq based footprinting analysis might more precisely measure its activity in specific cell types. Cell culture-based research in regard to MEOX1 function indicates senescence induction in endothelial cells. MEOX1 inhibition could thus reduce capillary rarefaction and be of benefit in cardiovascular disease.^4^ Redundant functions of MEOX1 and MEOX2 in endothelial cells might limit side effects.^4^ Transcription factors usually have broad effects on different cell functions. Besides being anti-fibrotic, inhibiting MEOX1 might also influence other cell functions. More studies are needed to further investigate the role of MEOX1 in endothelial cells and myofibroblasts to estimate potential side effects.

In summary, Alexanian et al. has demonstrated that the single-cell-based investigation of cell states in diseased tissue, combined with the controlled perturbation of transcription signaling, is a powerful tool to identify novel cell-specific targets for targeted therapy in fibrotic disease. Their study uncovered the MEOX1-dependent transcriptional switch from fibroblast to pro-fibrotic myofibroblast in heart failure and cardiac fibrosis. The upregulation of MEOX1 in myofibroblasts of different organs indicates the potential of MEOX1 inhibition in regard to fibrotic disease. Even though the development of drugs targeting transcription factors is challenging, the recent development of new strategies could be useful in targeting MEOX1.
